# The Usefulness of Soluble ST2 Concentration in Heart Failure with Reduced Ejection Fraction to Predict Severe Impairment in Exercise Capacity Assessed in Cardiopulmonary Exercise Testing

**DOI:** 10.3390/biomedicines13010060

**Published:** 2024-12-30

**Authors:** Magdalena Dudek, Marta Kałużna-Oleksy, Filip Sawczak, Agata Kukfisz, Aleksandra Soloch, Jacek Migaj, Maciej Lesiak, Ewa Straburzyńska-Migaj

**Affiliations:** 1 1st Department of Cardiology, Poznan University of Medical Sciences, 61-701 Poznań, Poland; marta.kaluzna@wp.pl (M.K.-O.); fsawczak@gmail.com (F.S.); ola.soloch@gmail.com (A.S.); jacek.migaj@gmail.com (J.M.); maciej.lesiak@usk.poznan.pl (M.L.); ewa.straburzynska-migaj@usk.poznan.pl (E.S.-M.); 2 3rd Department of Cardiology, Silesian Center for Heart Disease, Faculty of Medical University of Silesia, 41-800 Zabrze, Poland; agata.kukfisz@gmail.com

**Keywords:** ST2, sST2, biomarker, cardiopulmonary exercise testing, CPET, heart failure, heart failure with reduced ejection fraction, HFrEF

## Abstract

**Background/Objectives**: Heart failure (HF) constitutes a complex clinical syndrome that is highly prevalent worldwide, comprises a serious prognosis, and results in a reduced quality of life. Exercise capacity is one of the most significant parameters involved in the prognosis in HF patients. Our objective was to evaluate the relationship between the selected cardiopulmonary exercise testing (CPET) parameters and the concentration of novel biomarker sST2 in a group of patients with heart failure with reduced ejection fraction (HFrEF). **Methods**: A group of 135 patients with HFrEF was enrolled in this prospective cohort study. Patients were in the stable phase of the disease in the prior 4 weeks and received optimal medical treatment. Clinical and biochemical parameters were investigated. All patients performed maximal CPET. **Results**: The mean (SD) concentration of sST2 was 45.5 ± 39.2 ng/mL. Based on the CPET results, the cut-off value (52.377 ng/mL) was established, optimal for the discrimination of relative peakVO_2_ < 12 mL/kg/min. Patients were divided into two groups according to sST2 cut-off values determined with an ROC curve (AUC 0.692, 95% CI: 0.567–0.816). The mean relative peakVO_2_ in patients with higher sST2 was 14.5 ± 4.6 mL/kg/min, while in the second group, it was 17.6 ± 5.2 (*p* = 0.002). In the sST2 ≥ 52.377 ng/mL group, 55.6% of patients achieved VO_2_ < 50%. Subjects with lower sST2 values obtained higher values of PETCO_2_ (*p* < 0.001) and higher values of pulse O_2_ (*p* = 0.01). VE/VCO_2_slope (*p* = 0.002) was higher in patients with increased sST2 concentration. **Conclusions**: The concentration of sST2 protein is substantially associated with the clinical severity of heart failure with reduced left ventricular ejection fraction assessed by functional capacity through CPET.

## 1. Introduction

Heart failure (HF) represents a complex clinical syndrome that is highly prevalent worldwide [1]. The hallmark of chronic heart failure (CHF) is exercise intolerance, i.e., an impaired ability to perform physical activity with accompanying symptoms of significant shortness of breath and fatigue, which is associated with a reduced quality of life and increased mortality [2]. The pathophysiological mechanisms underlying exercise intolerance in HF are complex and include the impairment of cardiac and pulmonary reserves, as well as a reduced peripheral and respiratory perfusion of skeletal muscles.

Exercise capacity constitutes one of the most vital parameters related to prognosis in HF patients. Peak oxygen uptake (VO_2_), measured during maximal progressive physical exertion with the use of cardiopulmonary exercise testing (CPET), is the gold standard for determining exercise capacity in patients with HF [3] and therapy modifications [4]. CPET is a non-invasive, objective simultaneous measurement of cardiovascular and respiratory function during exercise, performed to assess exercise capacity in healthy subjects, as well as in patients with CHF. Multiple CPET parameters are predictors of mortality in HF patients, although several studies indicate that peak VO_2_ is the most crucial variable [5,6]. VO_2_max may be expressed as absolute values (L/min) and relative to total body mass values (mL/kg/min) [7]. Furthermore, other CPET test parameters have become increasingly emphasized, such as the value of the increased exercise ventilation index (VE/VCO_2_ slope), end-expiratory carbon dioxide pressure (PETCO_2_) or oxygen pulse. These, in turn, correlate with the prognosis in this group of patients, according to a number of data derived from the analysis of respiratory gases and hemodynamic measurements performed during exercise [6,8]. In terms of evaluating patients with heart failure, a comprehensive assessment of patients using CPET in combination with new biomarkers has recently been greatly highlighted.

According to the 2021 European Society of Cardiology Guidelines for the diagnosis and treatment of acute and chronic HF, relative peak VO_2_ ≤ 12 mL/kg/min defines a severe impairment of exercise capacity with an inability to exercise and represents one of the criteria for diagnosing advanced heart failure [2]. Patients who reached relative peak VO_2_ ≤ 12 mL/kg/min during CPET should be considered for qualification for heart transplantation [5].

The ST2 (suppression of tumorigenicity 2) protein has been increasingly explored as a potential biomarker in cardiovascular disease. ST2 is involved in the immune response and is secreted in response to the mechanical overload of the myocardium, thus providing information on myocardial remodeling and fibrotic processes [9,10]. Moreover, the IL-33/ST2L system is involved in cardioprotection, preventing fibrosis, hypertrophy and the apoptosis of cardiomyocytes, and it inhibits the inflammatory response [11]. It is of note that the association between sST2 and mortality rates has been confirmed in patients with heart failure with reduced ejection fraction (HFrEF) [10,12,13]. There are single reports available concerning the sST2 biomarker and the evaluation of physical capacity using CPET. Some studies have demonstrated a significant correlation between sST2 protein levels and CPET parameters, such as VO_2_ max [14,15]. Nevertheless, studies evaluating the relationship between sST2 and CPET parameters in HF patients are scarce.

The objective of this study was to assess the relationship between the selected CPET parameters and sST2 concentration in a group of patients with HFrEF.

## 2. Materials and Methods

### 2.1. Study Population

In this cross-sectional study, patients with heart failure with reduced ejection fraction (HFrEF) who were hospitalized for the evaluation of heart failure in the cardiology department were enrolled. The inclusion criteria were as follows: left ventricular ejection fraction (LVEF) ≤ 40%, age ≥ 18 years, stable disease state (no hospitalization due to exacerbation/decompensation or need for intravenous diuretics for HF in the previous 4 weeks), and optimal pharmacological treatment according to the European Society of Cardiology guidelines for heart failure [2]. The exclusion criteria were heart failure caused by a primary valvular defect, hypertrophic cardiomyopathy, end-stage chronic kidney disease requiring dialysis therapy, inflammatory diseases, severe chronic obstructive pulmonary disease (GOLD 4), and intellectual impairment making cooperation impossible. Additionally, the participants were required to sign the informed consent form. This study was conducted according to the Declaration of Helsinki guidelines and approved by the local Bioethics Committee (approval number 378/19).

### 2.2. Analyzed Parameters

The collected data included age, gender, comorbidities, etiology of HF, New York Heart Association (NYHA) functional class, prescribed medications, echocardiography assessment and CPET results. We analyzed complete blood count and other laboratory parameters, i.e., sST2, natriuretic peptides, creatinine, aspartate, electrolytes (sodium, potassium), creatinine and high-sensitivity C-reactive protein (hsCRP).

The sST2 concentration from the peripheral blood was performed using the ELISA method using the Presage^®^ ST2 Assay (Critical Diagnostics, San Diego, CA, USA) [16]. CPET was conducted using the Vmax29 Sensor Medics measurement module. Patients performed a symptom-limited maximal exercise test on a treadmill, either according to the RAMP protocol (load increment with a change in treadmill incline and speed was 0.5 MET/min), or according to the Bruce protocol modified for HF (an introductory step of 3 min with a treadmill speed of 1.7 km/h and an incline of 5% was added to the standard Bruce protocol). The left ventricular ejection fraction was calculated using the echocardiographic Simpson’s method in accordance with the European Society of Cardiology guidelines [17].

### 2.3. Statistical Analysis

Continuous variables are presented as mean ± standard deviation or median (25th percentile of the data—75th percentile of the data), depending on the presence of a normal distribution. Categorical variables are presented as numbers of subjects and corresponding percentages in parentheses. Normal distribution was tested with the Kolmogorov–Smirnov test. Patients were divided according to the optimal cut-off value of sST2 for the prediction of relative peak VO_2_ below 12 mL/kg/min. The Student’s *t*-test, Mann–Whitney U-test, and Chi^2^ test (with Yates correction if necessary) were performed where appropriate. Spearman’s non-parametric correlations were used to assess the association of sST2 with CPET findings. A Receiver Operator Characteristic (ROC) curve analysis of sST2 was employed to select the optimal cut-off value for the prediction of the relative peak VO_2_ below 12 mL/kg/min with the Youden method. A *p*-value of <0.05 was considered significant. All statistical analyses were conducted using STATISTICA 13.3 Tibco Software Inc., Palo Alto, CA, USA.

## 3. Results

The study involved 135 patients, among whom 91.9% were men. The mean (SD) age was 53.2 ±10.9 years, and the mean (SD) BMI was 28.5 ± 4.8 kg/m^2^. The most common comorbidities included arterial hypertension (48.2%), atrial fibrillation (AF) (37.8%), diabetes mellitus (28.2%) and chronic kidney disease (14.8%). The mean LVEF was 23.7 ± 8.1%. In the studied group, 67 patients presented with NYHA class III and ambulatory IV (49.6%). Most of the patients were administered the optimal HFrEF treatment at the time they entered the study.

**Figure 1:**
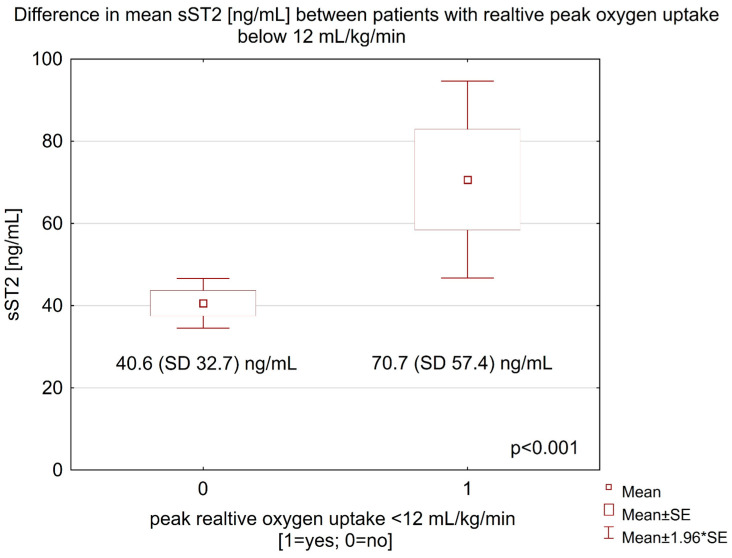
Difference in mean sST2 between patients with relative peakVO_2_ < 12 mL/kg/min.

**Figure 2:**
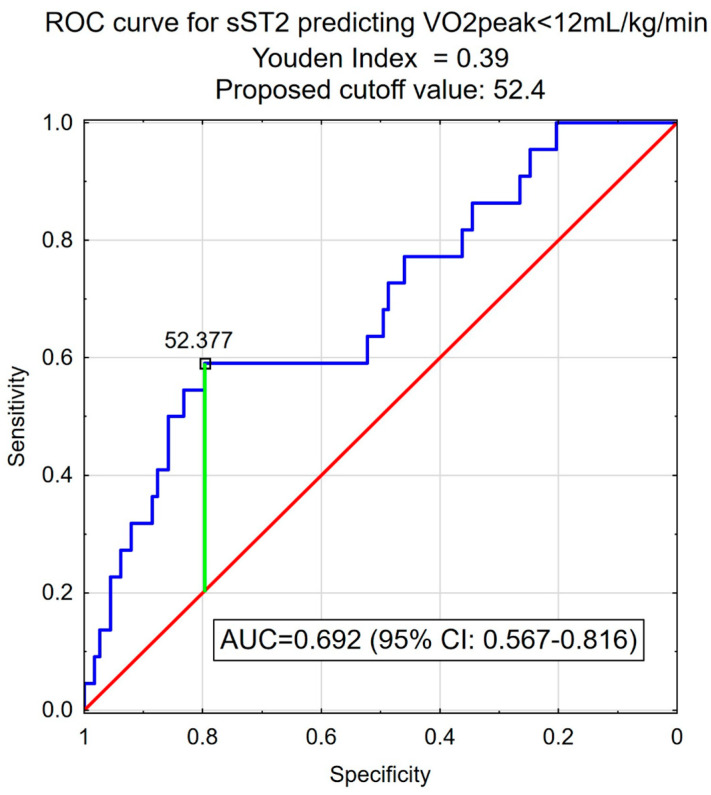
ROC curve for soluble ST2 predicting relative peakVO_2_ < 12 mL/kg/min. Abbreviations: ST2, suppression of tumorigenicity 2; peakVO_2_, peak oxygen uptake.

All patients who were enrolled in the study performed symptom-limited CPET, and sST2 concentration was measured in all patients. The mean (SD) concentration of sST2 was 45.5 ± 39.2 ng/mL. For the value of relative peak VO_2_ below 12 mL/kg/min sST2 concentration (70.7 ± 57.4 vs. 40.6 ± 32.7 ng/mL, *p* < 0.001) was significantly higher (Figure 1). Based on the CPET results, the sST2 cut-off value (52.377 ng/mL) optimal for the discrimination of relative peak VO_2_ < 12 mL/kg/min was established. Patients were divided into two groups according to the sST2 cut-off value determined with the ROC curve (AUC 0.692, 95% CI: 0.567–0.816) (Figure 2).

Patients in the group with sST2 concentration ≥ 52.377 ng/mL showed lower LVEF, a higher heart rate (HR) on discharge (*p* = 0.002), and more frequently AF (*p* = 0.01). Moreover, patients with a higher sST2 demonstrated more advanced symptoms of heart failure assessed in NYHA class (NYHA III or ambulatory IV class) (69.4% vs. 42.4%, *p* = 0.005 respectively). There was no significant difference in the HF etiology between the groups. Significantly higher levels of brain natriuretic peptide (BNP) (*p* < 0.001) and N-terminal pro-brain natriuretic peptide (NT-proBNP) (*p* < 0.001) were observed in patients with higher sST2. In addition, in this group, the serum albumin (*p* = 0.013) and sodium concentration were significantly lower (*p* = 0.005), and uric acid concentration was significantly higher (*p* = 0.04) (Table 1).

When dividing patients according to the sST2 level, we found a significantly lower forced vital capacity (*p* = 0.012) and a lower forced expiratory volume in the first second (*p* = 0.034) in patients with an sST2 concentration ≥ 52.377 ng/mL. The mean peak oxygen consumption in the group of patients with higher sST2 was 14.5 ± 4.6 mL/kg/min, while in the other group it was 17.6 ± 5.2 (*p* = 0.002). In the sST2 ≥ 52.377 ng/mL group, 55% of patients achieved a VO_2_ below 50% of the age- and sex-specific reference value, whereas in the other group, it was equal to 35.4% (*p* = 0.035).

Furthermore, other parameters of CPET differed significantly between the two groups. Patients with lower sST2 protein values obtained higher values of PETCO_2_ (*p* < 0.001), and lower values of oxygen pulse were found in the same group (*p* = 0.011). Differences were also demonstrated in the VE/VCO_2_ slope (*p* = 0.002), which was significantly higher in subjects with higher sST2 concentration. The participants with lower levels of sST2 presented higher maximum systolic blood pressure (*p* = 0.017) and diastolic blood pressure (*p* = 0.04) during CPET (Table 2).

**Figure 3:**
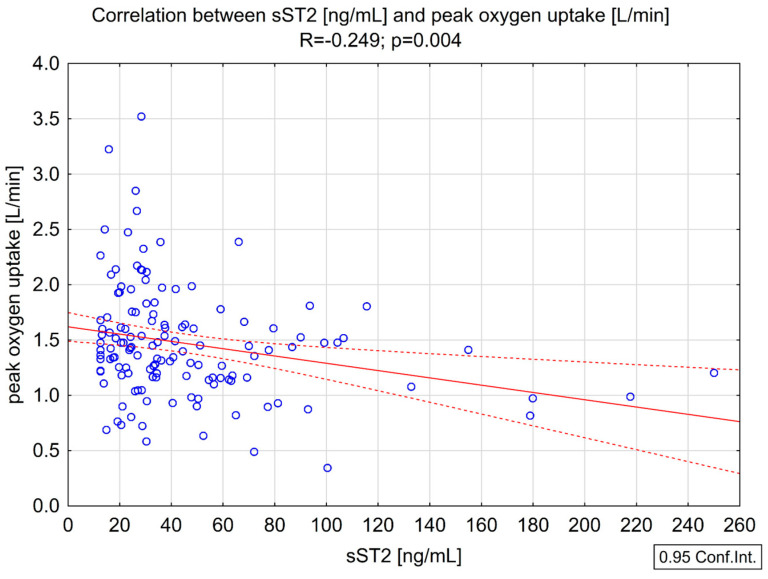
Correlation between sST2 and peakVO_2_.

**Figure 4:**
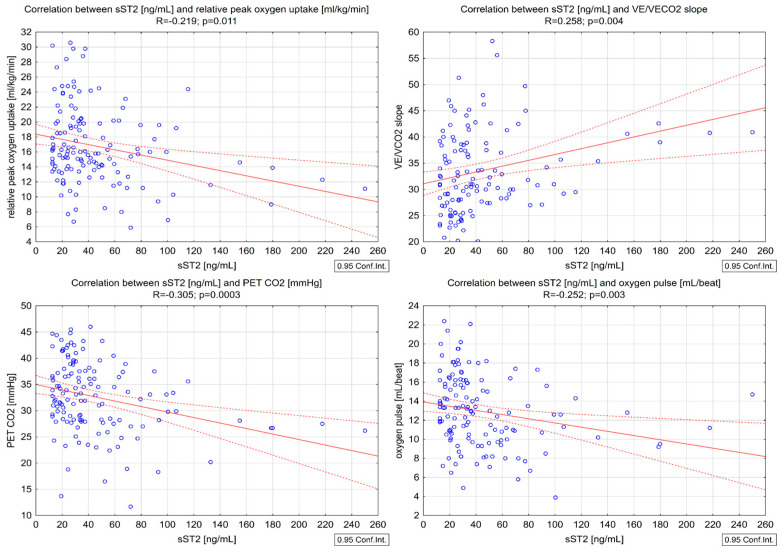
Correlations between sST2 and relative peakVO_2_, VE/VCO_2_, PETCO_2_, and oxygen pulse.

We found significant negative correlations between sST2 and peakVO_2_ (R = −0.249, *p* = 0.004) (Figure 3), relative and peak VO_2_ (R = −0.219, *p* = 0.011), oxygen pulse (R = −0.252, *p* =0.003), and PETCO_2_ (R = −0.305, *p* < 0.001) (Table 3), as well as a positive correlation with the VE/VCO_2_ slope (R = 0.258, *p* = 0.004) (Figure 4).

## 4. Discussion

CPET is an essential tool used to assess the functional capacity of HF patients and in qualifying them for heart transplantation [2,5]. However, it may be difficult to access in the clinical setting, and the majority of scientific papers employ the NYHA scale to assess patients’ physical performance, which may be subjective. Felker et al. [15] found a higher NYHA class in subjects with higher sST2 protein concentrations. Our study assessed the relationship between sST2 and physician-assessed functional capacity (NYHA class), and it objectively measured the maximum functional capacity in CPET. To our knowledge, there are very few prospective studies that assess the correlation between sST2 and CPET parameters. Our study is first to propose a cut-off value for sST2 for prediction of advanced heart failure assessed with CPET.

In the presented study, sST2 was demonstrated to be significantly correlated with CPET parameters in the group of HF patients. Moreover, we also found that patients with a higher sST2 concentration showed a higher NYHA class.

In the analyzed sub-population of the HF-ACTION study [15], a significant correlation was found between the sST2 concentration and both the clinical assessment using the NYHA classification and objective measurements of functional capacity during CPET, which is consistent with our observations. In the current study, as in the study by Felker et al. [15], we found significantly lower values of peak VO_2_ and higher values of VE/VCO_2_ in patients with higher sST2 concentrations. Although in both studies all patients suffered from HFrEF, it is worth bearing in mind that there were several crucial differences between the study conducted by Felker et al. [15] and our study presented here. The study group in their research was significantly larger (n = 912) and primarily consisted of men. Moreover, the patient population of the study by Felker et al. [15] was more diverse in terms of the NYHA classification (NYHA II—63.4%, NYHA III—35.6%, NYHA IV—1%), and patients were older (median 59.2 years, IQR 51.1–68.0) than in our research. We would like to emphasize that in the HF-ACTION study, only two parameters from CPET were analyzed.

The CPET parameters analysis showed differences for forced expiratory volume in 1 **s** (FEV1) (*p* = 0.03) and forced vital capacity (FVC) (*p* = 0.01) between the groups divided according to sST2 levels; however, no significant difference was found in the FEV1/FVC index. These differences are likely to stem from a more advanced heart failure and the possible coexistence of a restrictive spirometry pattern (RSP) in patients with higher levels of sST2. The Jackson Heart study [18] showed that the baseline RSP was more common in patients who were subsequently hospitalized for HF. Furthermore, a high incidence of restrictive-type ventilation disorders was also observed in patients with HF, which constitutes a predictor of poor prognosis in patients eligible for heart transplant [19].

Only single reports regarding the determination of the sST2 biomarker and the assessment of physical capacity using CPET in patients with CHF are available. These studies retrospectively assessed the association between new biomarkers and exercise capacity as evaluated by CPET in patients with HFrEF and in subjects with heart failure with preserved ejection fraction [20]. They demonstrated a significant correlation between sST2 and only two parameters from CPET—peak VO_2_ and VE/VCO_2_ slope. Our study also found a significant correlation between sST2 and peak VO_2_ (R = −0.249, *p* = 0.004), relative peak VO_2_ (R = −0.219, *p* = 0.011), VE/VCO_2_ slope (R = 0.258, *p* = 0.004), oxygen pulse (R = −0.252, *p* = 0.003) and PETCO_2_ (R = −0.305, *p* < 0.001).

The relationship between the concentrations of new biomarkers and the parameters of the CPET in patients undergoing rehabilitation was studied by Billebeau et al. [14]. Their study showed higher sST2 concentrations in patients with lower peak VO_2_ values. Our analysis presented here is prospective study to confirm the existence of a correlation between the sST2 level and the results of the CPET study and seems to be the first study to propose a cut-off point for sST2 values for the prediction of advanced heart failure assessed as relative peak VO_2_ below 12 mL/kg/min. Moreover, we also observed significantly higher VE/VCO_2_ slope values in the group with sST2 values ≥ 52.377 ng/mL. Previous studies indicated that the VE/VCO_2_ slope values gradually increased with the disease progression [21]. Notably, this parameter allows for assessing the risk of cardiovascular events in patients with HF [22].

In the available sources, no data were found regarding the relationship between the sST2 protein concentration and other parameters of the spiroergometric test except for peak VO_2_ or the VE/VCO_2_ slope in patients with HFrEF. According to current reports, low PETCO_2_ values achieved in CPET were an independent factor correlating with a poorer prognosis of patients and a higher mortality in CHF patients [23]. In turn, our study showed that PETCO_2_ (*p* < 0.001) and oxygen pulse (*p* = 0.005) were significantly lower in the group of patients with a higher concentration of the sST2 biomarker. In the research by Oliveira et al. [24], the values of oxygen pulse for predicting cardiovascular events were analyzed. Age-predicted peak O_2_ pulse was demonstrated to be a strong and independent predictor of cardiac mortality and complemented peakVO_2_ in risk prediction in HF patients [25].

In comparison to the abovementioned studies, our analysis showed a significant association between the recognized prognostic parameters, the unconventional parameters from the spiroergometric test and the new biomarker sST2. Therefore, it seems that the sST2 protein in combination with measures of cardiopulmonary fitness may represent a valuable tool in the long-term treatment of patients with HF.

### Study Limitations

This is a single-center study involving a small patient group, which, nevertheless, was sufficient to demonstrate associations between the parameters of interest. However, the predictive power presented (AUC = 0.692) does not effectively predict CPET outcomes based on this single study, although high sST2 may help to identify patients with poor CPET outcomes. Nevertheless, this is a first step towards conducting further studies in this area on a larger group of patients. Women represented a small percentage of the study group. This was due to the inclusion of only HFrEF patients and the relatively young age of the subjects. In such groups, there is a much higher prevalence of men [26,27]. Females enrolled in high-income or upper middle-income countries in the Global Congestive Heart Failure registry were older than men [28]. Future research involving multi-center collaboration and larger, more diverse participant groups is essential to validate these findings and provide more comprehensive insights.

## 5. Conclusions

The concentration of the sST2 protein is significantly associated with the clinical severity of heart failure with reduced left ventricular ejection fraction and functional capacity assessed by cardiopulmonary exercise testing. Further studies including a larger number of patients are essential to confirm these data and to explore this important research topic in order to incorporate sST2 protein as a biomarker applied in the daily routine.

## Figures and Tables

**Table 1:** Baseline characteristics of the studied group. Comparison of the baseline characteristic parameters in two groups according to the sST2 cut-off value (52.377 ng/mL), optimal for the discrimination of relative peakVO_2_ < 12 mL/kg/min.

Characteristics	All (N = 135) ^a^	sST2 < 52.377 ng/mL (N = 99) ^a^	sST2 ≥ 52.377 ng/mL (N = 36) ^a^	*p* Value	
Age, years	53.2 (10.9)	53.4 (10.8)	52.7 (11.3)	0.72	
Females	11 (8.1%)	7 (7.1%)	4 (11.1%)	0.69	
IHD etiology	68 (50.4%)	50 (50.5%)	18 (50.0%)	0.96	
BMI, kg/m^2^	28.5 (4.8)	28.7 (4.4)	28.0 (6.0)	0.45	
LVEF, %	20.0 (20.0–30.0)	25.0 (20.0–30.0)	20.0 (15.0–25.0)	0.02	
Comorbidities					
DM	38 (28.2%)	26 (26.3%)	12 (33.3%)	0.42	
COPD	18 (13.3%)	13 (13.1%)	5 (13.9%)	0.86	
CKD	20 (14.8%)	12 (12.1%)	8 (22.2%)	0.24	
HA	65 (48.2%)	50 (50.5%)	15 (41.7%)	0.36	
AF	51 (37.8%)	31 (31.3%)	20 (55.6%)	0.01	
NYHA class					
I	4 (3.0%)	4 (4.0%)	0 (0%)	0.52	0.01
II	64 (47.4%)	53 (53.5%)	11 (30.6%)	0.02	
NYHA class III or IV	67 (49.6%)	42 (42.4%)	25 (69.4%)	0.005	
Biochemical parameters					
Hgb, mmol/L	9.06 (0.88)	9.06 (0.75)	9.06 (1.17)	0.43	
RBC, ×10^12^/L	4.79 (0.51)	4.74 (0.42)	4.93 (0.69)	0.38	
BNP, pg/mL	263.7 (109.8–468.0)	203.4 (86.3–367.9)	498.2 (385.5–839.9)	<0.001	
NT-proBNP, pg/mL	1466 (492–2526)	921 (403–1798)	2923 (1912–5319)	<0.001	
Uric acid, µmol/L	436.5 (117.7)	419.2 (96.1)	485.4 (155.8)	0.04	
Creatinine, µmol/L	103.0 (26.2)	100.0 (25.6)	111.3 (26.4)	0.03	
eGFR MDRD, mL/min/1.73 m^2^	74.3 (22.9)	77.0 (23.4)	66.7 (20.0)	0.02	
Serum protein, g/L	73.0 (8.4)	73.4 (9.1)	72.2 (6.3)	0.48	
Serum albumin, g/L	41.0 (3.4)	41.5 (3.3)	39.8 (3.5)	0.01	
TSH, mIU/L	1.51 (0.84–2.46)	1.51 (0.83–2.41)	1.65 (0.87–2.81)	0.15	
Na+, mmol/L	140 (138–141)	140 (138–141)	138 (136–141)	0.00453	
hsCRP, mg/L	2.27 (1.00–4.38)	1.80 (0.90–3.74)	5.05 (2.72–10.5)	<0.001	
Fasting glucose, mmol/L	5.97 (5.36–6.57)	5.88 (5.36–6.57)	6.05 (5.38–6.67)	0.70	
Medications					
Loop diuretics, %	123 (91.1%)	87 (87.9%)	36 (100%)	0.06	
β-blockers, %	133 (98.5%)	98 (99.0%)	35 (97.2%)	0.96	
ACEI/ARB, %	110 (81.5%)	83 (83.8%)	27 (75.0%)	0.24	
ARNI, %	18 (13.3%)	13 (13.1%)	5 (13.9%)	0.86	
MRA, %	120 (88.9%)	86 (86.9%)	34 (94.4%)	0.35	

Abbreviations: ACEI, angiotensin-converting enzyme inhibitor; AF, atrial fibrillation (paroxysmal, permanent or persistent); ARB, angiotensin receptor blocker; ARNI, angiotensin receptor neprilysin inhibitor; BMI, body mass index; BNP, B-type natriuretic peptide; CKD, chronic kidney disease; COPD, chronic obstructive pulmonary disease; DM, diabetes mellitus; eGFR MDRD, glomerular filtration rate estimated by the Modification of Diet in Renal Disease (MDRD); HA, arterial hypertension; Hgb, hemoglobin; hsCRP, high-sensitivity C-reactive protein; IHD, ischemic heart disease; LVEF; left ventricular ejection fraction; MRA, mineralocorticoid receptor antagonist; NT-proBNP, N-terminal pro-B-type natriuretic peptide; NYHA, New York Heart Association Classification; RBC, red blood count; TSH, thyroid-stimulating hormone. ^a^ Data presented as XX (YY) do not follow a normal distribution (XX—mean, YY—standard deviation), whereas data presented as XX (YY–ZZ) do not follow a standard distribution (XX—median, YY—lower quartile, ZZ—higher quartile).

**Table 2:** Comparison of cardiopulmonary exercise testing parameters in two groups according to sST2 cut-off value (52.377 ng/mL) optimal for the discrimination of relative peak VO_2_ < 12 mL/kg/min.

CPET Parameter	All (N = 135) ^a^	sST2 < 52.377 ng/mL (N = 99) ^a^	sST2 ≥ 52.377 ng/mL (N = 36) ^a^	*p* Value
%VO_2_ < 50%	55 (40.7%)	35 (35.4%)	20 (55.6%)	0.04
FVC, L	3.72 (0.94)	3.84 (0.97)	3.38 (0.74)	0.01
FEV1, L	2.77 (0.78)	2.86 (0.81)	2.54 (0.64)	0.03
FEV1/FVC	74.9 (10.9)	74.7 (11.0)	75.3 (10.7)	0.77
relative peak VO_2_, mL/kg/min	16.8 (5.2)	17.6 (5.2)	14.5 (4.6)	0.002
peak VO_2_, L/min	1.47 (0.51)	1.55 (0.52)	1.24 (0.40)	0.001
relative peak VO_2_ < 12 mL/kg/min, %	22 (16.3%)	9 (9.1%)	13 (36.1%)	<0.001
%VO_2_pred, %	53.5 (16.1)	55.8 (15.6)	47.1 (15.8)	0.005
VE/VCO_2_ slope	33.6 (8.7)	32.3 (7.2)	37.7 (11.2)	0.002
O_2_ pulse, mL/beat	13.0 (10.5–15.9)	13.4 (10.8–16.2)	11.0 (9.5–13.5)	0.01
HR max, beats/min	117.1 (24.6)	118.6 (23.7)	112.7 (26.8)	0.23
SBP at rest, mmHg	110 (100–120)	110 (100–120)	109 (100–120)	0.16
DBP at rest, mmHg	70 (60–80)	70 (60–80)	60 (60–70)	0.07
SBP max, mmHg	140 (120–152)	140 (120–160)	130 (110–140)	0.02
DBP max, mmHg	80 (70–80)	80 (80–85)	77 (70–80)	0.04
PETCO_2_, mmHg	32.6 (6.9)	34.1 (6.4)	28.4 (6.6)	<0.001

Abbreviations: DBP, diastolic blood pressure; FEV1, forced expiratory volume in 1 s; peak VO_2_, oxygen uptake at peak exercise; FVC, forced vital capacity; HR, heart rate; O_2_ pulse, oxygen pulse; peak VO_2_ < 12 mL/kg/min, percent of patients with oxygen uptake at peak exercise below 12 mL/kg/min; SBP, systolic blood pressure; PETCO_2_, end tidal carbon dioxide partial pressure; VEVCO_2_ slope, minute ventilation-to-carbon dioxide output; VE, minute ventilation; %VO_2_pred, percent predicted oxygen uptake at peak exercise; %VO_2_ < 50%, percent of patients with predicted oxygen uptake at peak exercise below 50% predicted value. ^a^ Data presented as XX (YY) follow a normal distribution (XX—mean, YY—standard deviation), and data presented as XX (YY–ZZ) do not follow a normal distribution (XX—median, YY—lower quartile, ZZ—higher quartile).

**Table 3:** Spearman nonparametric correlation of sST2 with parameters of cardiopulmonary exercise testing.

CPET Parameter Correlated with sST2	Spearman R	*p*
%VO_2_ < 50% [%]	0.132	0.127
relative VO_2_peak [mL/kg/min]	−0.219	0.011
VO_2_peak [L/min]	−0.249	0.004
relative VO_2_peak < 12 mL/kg/min [%]	0.246	0.004
%VO_2_pred [%]	−0.204	0.018
VE/VCO_2_ slope	0.258	0.004
VE [L]	−0.055	0.529
O_2_ pulse [mL/beat]	−0.252	0.003
HR max [beats/min]	−0.061	0.481
SBP at rest [mmHg]	−0.050	0.611
SBP max [mmHg]	−0.134	0.146
PET CO_2_ [mmHg]	−0.305	0.0003

Abbreviations: %VO_2_ < 50%—percent of patients with predicted oxygen uptake at peak exercise below 50% prediction value, VO_2_peak—oxygen uptake at peak exercise, VO_2_peak < 12 mL/kg/min—percent of patients with oxygen uptake at peak exercise below 12 mL/kg/min, %VO_2_pred—percent predicted oxygen uptake at peak exercise, VE/VCO_2_ slope—minute ventilation-to-carbon dioxide output, VE—minute ventilation, O_2_ pulse—oxygen pulse, HR—heart rate, SBP—systolic blood pressure, PET CO_2_—end-tidal carbon dioxide partial pressure.

## Data Availability

The data presented in this study are available on request from the corresponding author.
